# The Use of a Quadripolar Left Ventricular Lead Increases Successful Implantation Rates in Patients with Phrenic Nerve Stimulation and/or High Pacing Thresholds Undergoing Cardiac Resynchronisation Therapy with Conventional Bipolar Leads

**DOI:** 10.1016/s0972-6292(16)30605-2

**Published:** 2013-03-07

**Authors:** Marc-Alexander Ohlow, Bernward Lauer, Michele Brunelli, Yunis Daralammouri, Christoph Geller

**Affiliations:** 1 Department of Cardiology, Zentralklinik Bad Berka, Robert-Koch-Allee 9, 99437 Bad Berka, Germany; 2 Department of invasive Electrophysiology, Zentralklinik Bad Berka, Robert-Koch-Allee 9, 99437 Bad Berka, Germany

**Keywords:** Threshold, phrenic nerve stimulation, quadripolar, lead, cardiac resynchronisation therapy

## Abstract

**Background:**

Phrenic nerve stimulation (PNS) and high pacing thresholds (HPT) hinder biventricular stimulation in patients (pts) undergoing cardiac resynchronization therapy (CRT). A new quadripolar left ventricular (LV) lead (Quartet 1458Q, St. Jude Medical) with increased number of pacing configuration, might overcome this problem.

**Methods:**

All consecutive pts in whom a standard bipolar lead intraoperatively resulted in PNS and/or HPT (≥4.00V/1mV), received, during the same implant, a quadripolar LV lead. Aim of the study was to evaluate acute and short term outcome.

**Results:**

26 pts [24 (92%) male, mean age 74±6 years)] with PNS (22 pts; 85%) and HPT (4 pts; 15%) were included. Permanent right ventricular pacing was the reason for broad QRS complex in 4 (15%) pts, whereas all other pts had a left bundle branch block. Severely symptomatic (NYHA Class ≥3) heart failure with reduced ejection fraction (EF 31±9%) was mostly caused by ischemic heart disease (14 pts; 54%). Idiopathic dilated cardiomyopathy and valvular heart disease were diagnosed in 6 (23%) pts each. In most (24/26, 92%) pts the use of the Quartet lead led to successful biventricular pacing due to a significant reduction in intraoperative pacing threshold (5.2V/1.0ms vs. 1.4V/0.8ms; p=0.03), which was maintained (1.2V/0.7ms) at follow-up. PNS never represented reason for failed LV pacing, neither acutely nor during follow-up.

**Conclusion:**

Excessively HPT and/or PNS are frequently encountered when conventional bipolar leads are used for CRT. A new quadripolar LV lead increases the rate of successful biventricular stimulation. Lower pacing threshold and freedom from PNS are maintained at follow-up.

## Introduction

Cardiac resynchronization therapy (CRT) reduces hospitalization, morbidity, and mortality in patients with left bundle branch block and left ventricular (LV) dysfunction [1-3]. Nevertheless, failure to pace the LV occurs in up to 15% of implants [4-6]. Excessively high pacing thresholds (HPT), phrenic nerve stimulation (PNS) as well as inability to cannulate the coronary sinus (CS) ostium or to enter the target vein with the pacing lead reduce the rate of successful biventricular pacing [4-6]. Programmable multiple pacing configurations increases the rate of effective LV stimulation, reducing the rate of excessively HPT and PNS [7,8]. The Quartet (1458Q; St Jude Medical, Sylmar, CA, USA) lead, a multipolar LV lead with three ring electrodes in addition to the tip electrode, has been designed with this purpose. All consecutive patients, in whom a standard bipolar lead intraoperatively resulted in PNS and/or HPT (≥4.00V/1mV), received, during the same implant, a quadripolar LV lead. Aim of the study was to evaluate acute and short term outcome.

## Methods

### Patient population

Between July 2010 and October 2011, all consecutive patients in whom standard bipolar LV lead resulted in intraoperative excessively HPT (≥4.0V/1.0ms) and/or PNS (i.e. diaphragmatic contraction at fluoroscopy simultaneous with the pacing artefact), which could not be overcome by at least 2 attempts of repositioning, were enrolled in this prospective observational study. During the index procedure, the standard bipolar lead was removed and the Quartet lead was positioned in the same vein. Indication for cardiac resynchronization therapy with cardioverter defibrillator (CRT-D) followed the guideline of the European Society of Cardiology [9,10]. The study protocol was approved by the institutional ethics committee.

### Study devices

**Figure 1:**
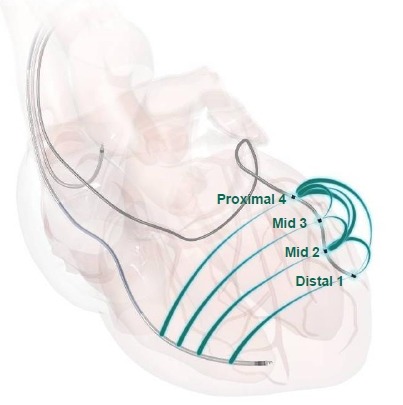
Ten vectors available using the four electrodes on the Quartet lead and the right ventricular (RV) coil: 1. distal 1 (D1) to mid 2 (M2); 2. D1 to proximal 4 (P4); 3. D1 to RV coil; 4. M2 to P4; 5. M2 to RV coil; 6. mid 3 (M3) to M2; 7. M3 to P4; 8. M3 to RV coil; 9. P4 to M2; and 10. P4 to RV coil (image courtesy of St. Jude Medical)

The Quartet Model 1458Q is a new quadripolar lead designed to pace the left ventricle from the CS venous system (Figure 1). The Quartet is a pre-shaped, quadripolar electrode, over-the-wire LV lead that can accommodate the use of either a stylet or a guidewire and is available in lengths of 75, 86, or 92 cm.

The LV lead body has a maximum diameter of 4.7F, while the four titanium nitride-coated platinum-iridium alloy electrodes are 5.1F. The three ring electrodes are located 20, 30, and 47 mm from the distal tip electrode. The lead is insulated with Optim™ insulation. All four electrodes can be programmed as cathode, while the second and the most proximal (Mid 2 and Proximal 4) can be programmed as anode. This, together with the coil of the right ventricular (RV) shocking lead, which may act as anode, offers 10 bipolar pacing configurations (Figure 1). These include 6 bipolar configurations (i.e. both cathode and anode are located on the Quartet lead) and 4 extended bipolar configurations (i.e. the RV coil is used as the anode electrode). In this study, the Quartet lead was connected to the Promote Q (model CD3221-36) cardiac resynchronization therapy-defibrillator (CRT-D) device (St Jude Medical), which is equipped with a new IS4 header.

### Lead Evaluation at Implantation and Follow Up

Implantation of any commercially available right atrial (RA) or RV pacing/defibrillation leads was allowed. At implant, lead impedance, signal amplitude, and capture threshold (at a pulse width of 0.5ms) were recorded for RA and RV leads. Following removal of the previously positioned bipolar lead, the Quartet LV lead was advanced in the same target vein and connected with a Promote Q CRT-D device. Testing of all LV lead vectors at 0.5 ms and 1.5 ms pulse width to determine the best pacing configuration, in addition to impedance and signal amplitude, were collected for the Quartet lead. Procedural and fluoroscopy time were recorded as well.

Before hospital discharge and at 1 month post-implant, LV capture thresholds and lead impedances were measured for all 10 pacing configurations. The presence of PNS, tested to the maximal output of 7.5V/0.5 ms, was determined for all 10 vectors at each visit. Programming of the LV pacing configuration was left to the judgement of the physician carrying out the device interrogation.

In order to compare outcomes between different pacing vectors, boundaries that define a successful pacing configuration were defined and set at a pacing threshold ≤2.5V/1ms without PNS at a maximal pacing output of 7.5 V/1ms.

### Statistical analysis

Categorical variables are presented as percentages or numbers. Continuous variables are summarized as mean ± standard deviation. Comparisons were made with Fisher's exact test for categorical variables. A Mann-Whitney U test was used to compare continuous variables. All tests were carried out with the statistic software SAS, version 9.2, and Stata data analysis software (Stata Corp. LP). A P-value <0.05 was considered statistically significant.

## Results

### Population characteristics

During the 16 months study period a total of 278 patients underwent cardiac resynchronisation therapy at our institution. Of those, 26 patients (9%) [24 (92%) male, mean age 74±6 years)] experienced PNS in 22 (85%) patients or HPT in 4 (15%) patients during the index procedure and represent the study population. The mean ejection fraction was 31±9%, and 14 patients had ischemic (54%) and six (23%) valvular heart disease etiology. Idiopathic dilated cardiomyopathy was diagnosed in 6 (23%) patients.

### Procedural data

The quadripolar lead could be advanced in the same vein of the coronary sinus in all 26 patients. In 20 (77%) of them the lead was positioned in the vein judged ideal at coronary sinus angiography.

However, in 2 (8%) patients excessively high pacing threshold (>2.5V/1.0ms) with each of the quadripolar lead stimulation vectors crossed the pre-defined limit for successful implantation. In the remaining 24 (92%) patients the mean intraoperative pacing threshold was 1.4±0.5V at 0.8±0.5ms (versus 5.2±1.7V/1.0±0.32ms; p=0.03 with conventional bipolar pacing). PNS could be avoided in all 22 patients in whom the quadripolar lead was used for this reason. In addition, PNS never represented reason for failure in patients in whom the quadripolar lead was implanted due to excessively HPT. In almost half of the patients (11/24, 42%) excessively HPT or PNS was overcome with a "standard" (8 patients=Distal 1 to RV Coil; 3 patients=Mid 2 to RV Coil) pacing configuration.

Additional placement of a quadripolar lead (due to excessively HPT/PNS) significantly prolongs the procedural time (153±39 min vs. 87±18 min; p=0.02) as well as the fluoroscopy time (18±13 min vs. 11±8; p=0.04). Procedural data are summarized in Table 1.

### Follow up

**Figure 2:**
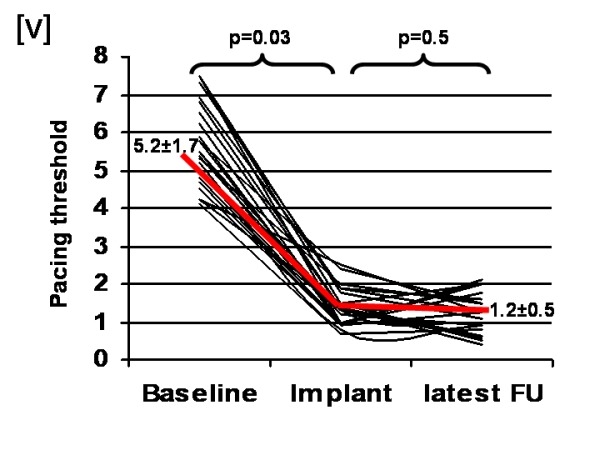
Pacing thresholds with conventional bipolar LV-leads (baseline), with quadripolar LV-leads (implant), and quadripolar LV-leads at follow-up (latest follow-up)

No patients were lost at follow up. The last CRT-D interrogation was at a mean of 5.2±5 months (range 1 month - 14 months) after the implantation. The LV-pacing threshold using the same configuration did not change between implantation and follow up (1.4±0.5V at 0.8±0.5ms vs. 1.2±0.5V at 0.7±0.3ms; p=0.5 - Figure 2).

The two patients (8%) with high pacing thresholds despite implantation of the quadripolar lead finally underwent surgical epicardial LV lead placement via minimal lateral thoracotomy during follow-up (pt #1 experienced dislocation of the quadripolar lead (1/26=3.9% dislocation rate), and pt #2 had a further increase of the pacing threshold).

## Discussion

In this series of consecutive patients undergoing CRT in whom implantation of a left ventricular bipolar lead invariably resulted in HPT and/or PNS, the use of a new quadripolar lead successfully overcame the problem in almost all of them.

CRT is an effective therapy that significantly reduces both morbidity and mortality [9,10]. Nevertheless failure to pace the LV occurs in up to 15% of implants [4-6] and clinically relevant PNS is observed either during implantation or follow up in up to 22% of the patients [11,12]. Our experience confirms these data, as 9% (26/278) of patients undergoing CRT had PNS and/or excessively HPT during the implantation, significantly limiting the number of patients actually receiving cardiac resynchronization therapy.

Lead repositioning in the same or in another coronary sinus branch, surgical implantation of an epicardial lead, downgrading the device from 3 to 2 chamber, and implantation of pacemaker leads offering more than one stimulation vector [13] are all appropriate solutions [12], but this prolongs implantation time without providing any security for success, requires a new (eventually in minimal thoracotomy) procedure or hinders cardiac resynchronization. A quadripolar lead (in combination with an appropriate CRT device) offering ten possible pacing vectors, has several theoretical advantages both at implantation and during follow-up [14,15], including the resolution of commonly encountered problems as excessively HPT and PNS.

This study has been able to confirm some of the theoretical advantages of this new quadripolar LV lead. The rate of effective cardiac resynchronization therapy reached almost 100% in a cohort of patients including complex cases some of whom undergoing redo implantations and in whom bipolar stimulation resulted in either excessively HPT or PNS after ≥2 attempts of lead reposition. This result seems even more relevant if it is considered that a significant proportion of patients were actually referred from other hospital due to impossibility to achieve effective CRT. Quadripolar LV lead application failed in only 2 of our 26 patients. In both of them an unacceptable pacing output was observed also with the use of a quadripolar lead. Quicker battery depletion and additional and progressive increase of the pacing output were the (certain and theoretical) reasons why both patients were referred for surgical LV-lead implantation.

Interestingly, in 42% of all patients in our series receiving quadripolar LV leads the best electrical performance could be obtained with "conventional" pacing vectors (D1 to M2, D1 to RV coil, and M2 to RV coil), which are available on bipolar LV leads as well. As already suggested, lead geometry and different inter-electrode spacing might have played a major role in giving this results [14,16]. The use of quadripolar electrode lead could assist operators in stimulating otherwise hard to reach proximal positions without compromising lead stability [16]. E.g. the pacing configuration "M2 to RV coil" (as used in 12% of our patients) is of course also available with conventional bipolar leads but if this pacing configuration has to be applied in the proximal parts of the target vein there might be some concerns about long-term lead stability. However, the authors have to concede, that subtle difference between bipolar and quadripolar lead position might play also a role in some of the cases with "D1 to RV coil" pacing configuration (as used in 30% of our patients).

The results obtained during the implantation were confirmed at subsequent device interrogation. Even though the follow up was relatively short (mean 5 months) PNS never appeared to be a problem, the pacing threshold hold stability, and, even if non-significantly, it actually decreased over time. Although one LV-lead dislodgement was observed, accounting for 3.9% of the patients, during the relatively short follow up, the displacement rate is similar to that seen with conventional bipolar lead LV leads [4,5,17]. Whether the increased lead stiffness given by the four electrodes will offer greater stability at a longer follow up, needs to be addressed.

In conclusion, doubling electrodes on the LV pacing lead seems offers additional advantages that seems stable over time. Whether additional increase in electrode numbers would further raise the likelihood of successful CRT needs to be proved. At the same time, even more electrodes might turn into excessive lead stiffness, leading to reduced maneuverability, and reduced lead longevity. In the future, technological advances might also provide the ability to pace the (left) ventricle from several electrodes at the same time, this might also results in increase CRT efficacy.

### Study limitations

This study has several limitations. It is a single-center, non-randomized study conducted in a relatively small number of patients. In addition, the follow-up is limited to a mean of 5 months post implantation and thus there might be late or unforeseen problems with this lead.

## Summary

Excessively HPT and/or PNS are frequently encountered when conventional bipolar leads are used for CRT. A new quadripolar LV lead increases the rate of successful biventricular stimulation. Lower pacing threshold and freedom from PNS are maintained at follow-up.

## Figures and Tables

**Table 1:**
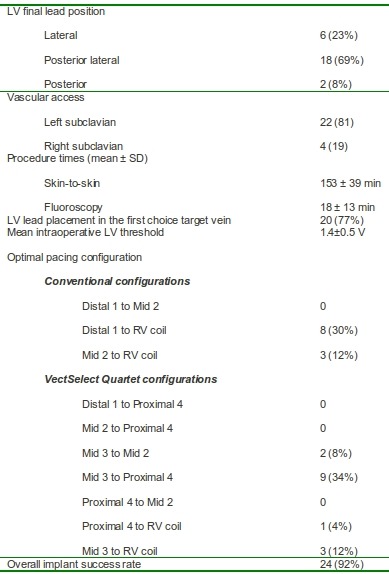
Procedural data
